# Supplementary material for: Excitability regulation in the dorsomedial prefrontal cortex during sustained instructed fear responses: a TMS-EEG study
Source: Sci Rep. 2018 Sep 28;8:14506. doi: 10.1038/s41598-018-32781-9 (PMC6162240; doi:10.1038/s41598-018-32781-9)
Supplement: Supplementary file 1 — Supplementary information [file 41598_2018_32781_MOESM1_ESM.docx]

**Excitability regulation in the dorsomedial prefrontal cortex during sustained instructed fear responses: a TMS-EEG study**

Gabriel Gonzalez-Escamilla^1^, Venkata C. Chirumamilla^1^, Benjamin Meyer^2^, Tamara Bonertz^1^, Sarah von Grotthus^1^, Johannes Vogt^3^, Albrecht Stroh^4^, Johann-Philipp Horstmann^5^, Oliver Tüscher^5^, Raffael Kalisch^2^, Muthuraman Muthuraman^1^, Sergiu Groppa^1^

^1^Section of Movement Disorders and Neurostimulation, Department of Neurology, Focus Program Translational Neurosciences (FTN), University Medical Center of the Johannes Gutenberg University Mainz, 55131 Mainz, Germany.

^2^Neuroimaging Center Mainz, Focus Program Translational Neuroscience, University Medical Center of the Johannes Gutenberg University Mainz, 55131 Mainz, Germany

^3^Institute for Microscopic Anatomy and Neurobiology, University Medical Center of the Johannes Gutenberg University Mainz, 55131 Mainz, Germany.

^4^Focus Program Translational Neurosciences, Institute for Microscopic Anatomy and Neurobiology, Johannes Gutenberg University Mainz, 55131 Mainz, Germany.

^5^Department of Psychiatry and Psychotherapy, University Medical Center of the Johannes Gutenberg University Mainz, 55131 Mainz, Germany.

**Supplementary information**

**Extended methods**

**EEG signal processing and analysis**

EEG signals without TMS-related artefact were then processed to account for line-noise (50 Hz) by means of the discrete Fourier transform (DFT) filter over 50 Hz frequencies. The DFT filter consist of a notch filter applied to the data in order to remove ('zeroing') the 50Hz line noise components, also including the harmonics at 100 and 150 Hz. This removal is done by fitting a sine and cosine at the specified frequency to the data and subsequently subtracting the estimated components. Following, linear trends were eliminated by fitting a low-order polynomial to the data and subtracting the fit. Both processes were performed using the built ft_preprocess function from the open source FieldTrip toolbox. EEG data was then further visually inspected on a trial-basis. The visual inspection of the data consisted of a quantitative display of the signal variance and maximum and minimum amplitude of each trial using the FieldTrip's ft_rejectvisual function. At each trial showing extreme variance and amplitudes, the segments containing noise were then marked and interpolated over time using the ft_interpolatenan function. Using the same approach channels with high variances were also marked and interpolated by spherical spline by interpolating all neighbor channels using the ft_channelrepair function. As result from the visual inspection no systematic muscular activity was observed in the channels. Thus no trials were removed/excluded from subsequent analyses. Such visual examination also allow that contaminated periods within trials to be fixed by means of interpolation.

After removal of the TMS pulse and correction of the trials with topographical interpolation (see above) we performed independent component analysis (ICA) correction (data not shown). However, we observed no clear difference (artifact-related components) of ICA over the non-ICA corrected signals. Thus ICA was not included in the final analyses, this decision was further a consensus from the observed results on current data and recent evidence of induced distortion in the evoked amplitudes when removing ICA components [^1^](#_ENREF_1)^,^[^2^](#_ENREF_2).

**TMS experiment without task stimuli**

As the comparison between ERP and TEP is not straightforward due the TMS-pulse, which induces activity in the target site and alters the EEG signals. An additional experiment to the main TMS experiment was acquired, this session included navigated TMS stimulation to the right dmPFC but without any task presentation, thus a pure TMS session. This session has the advantage of having 60 trials, which as mentioned above has been shown to produce robust TMS evoked potentials[^3^](#_ENREF_3).

We have used this dataset to provide evidence on the reliability of the presented activity peaks during the main TMS experiment. For this aim, and as mentioned in the “*EEG signal processing and analysis”* of the methods section, we computed the signal-to-noise-ratio (SNR) of the time-locked activity (evoked potentials), but this time in the no-task TMS session and using different number of trials each time.

The statistical comparison (repeated measures ANOVA) of the SNR between trials revealed no main effects of the used trial number (F = 0.69, p = 0.75), and the same for the post hoc analyses corrected with Tukey HSD test (all p > 0.05). An additional comparison was performed setting an ANOVA between the SNR of the TMS data (36 trials) and the TMS-without-task (35 to 60 trials). This analyses also showed no differences between the two experiments (supplementary Fig. 3) irrespective of the number of trials used in the TMS-without-task (F = 0.076, p = 0.93), evidencing the feasibility of detecting reliable evoked responses in our main TMS experiment.

Additionally, the analyses evidenced that the increased latencies and amplitudes in TEPs T-NT compared to ERP T-NT, which have been interpreted as functional differences, are not caused by the use of TMS on the main experiment, because the TMS without task clearly resembles the no-Threat condition in the TMS experiment with only small differences on the wave shape and amplitudes and diverges from the threat condition (supplementary Fig. 4). Here, the TEP197 showed significantly increased amplitude for NT TMS-evoked-activity compared to TMS-no-task (p = 0.018, T = 2.58). When contrasting the threat TMS-evoked-activity clearly remains away from both waves. The statistical comparison showed increased amplitudes for T TMS-evoked-activity compared to the no-task in almost all TEPs (p < 0.03, T > 2) expect for TEP81 (p = 0.07, T = 1.95) and TEP117 (p = 0.1, T = 1.7). Thus enabling to obtain a measure of how physiological potentials are modulated by dMPFC activity, while controlling for any unspecific TMS effects not related to cortical responses. With this data we show that the evoked activity of this session highly resembles the evoked activity of the no-threat, and while some TEP differences remain (likely to be TMS effects) others seem to be just related with the physiological responses modulated by the dmPFC.

**Supplementary Figure 1.** Regional aggrupation of the EEG sensors according to the underlying brain regions (lobules).


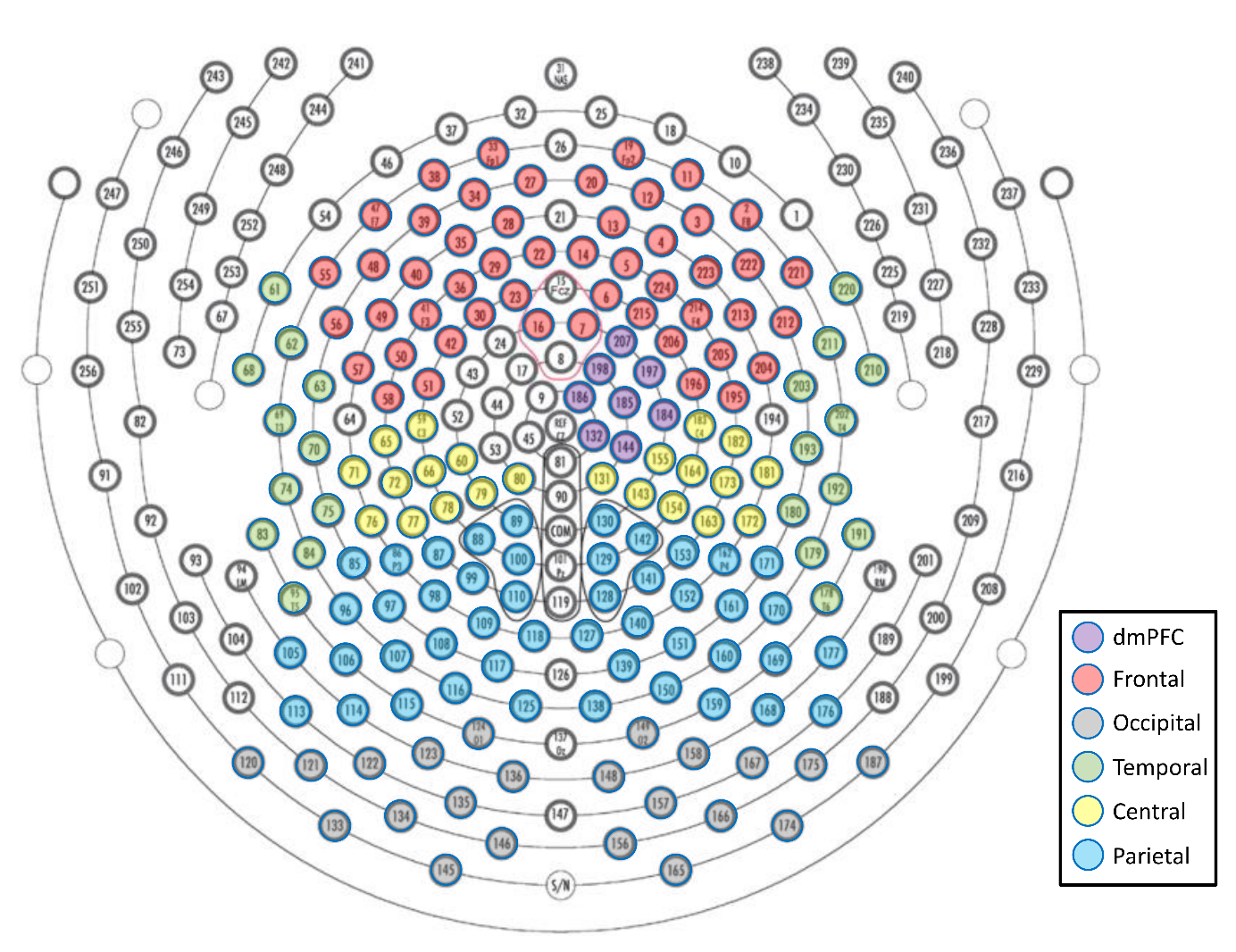


**Supplementary Figure 2.** Parcellation of the medial prefrontal cortex (mPFC) according to Etkin et al.[^4^](#_ENREF_4) Abbreviations: ACC, anterior cingulate cortex; sg, subgenual; pg, pregenual; vm, ventromedial; rm, rostromedial; dm, dorsomedial; ad, anterior dorsal; pd, posterior dorsal.


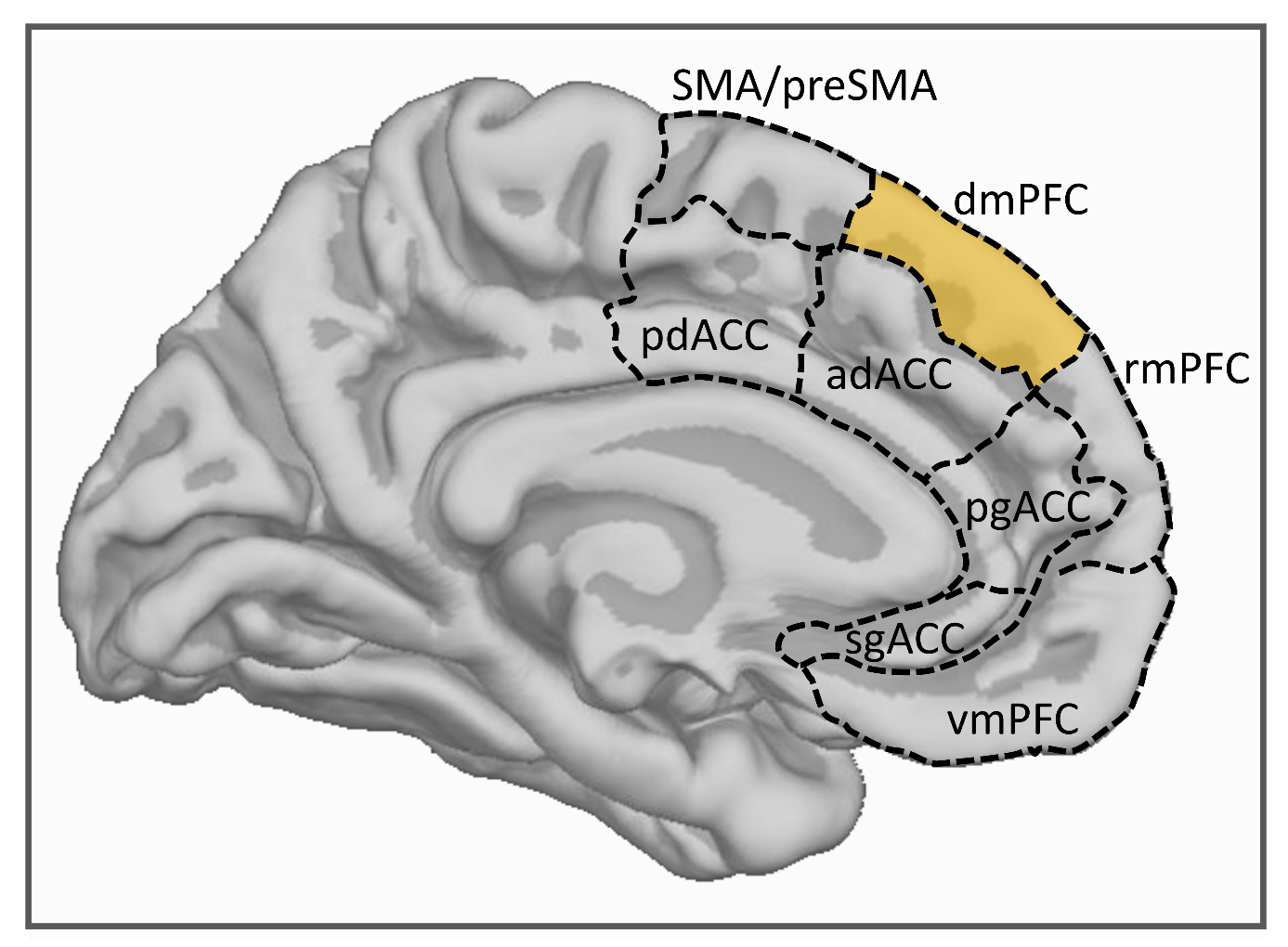


**Supplementary Figure 3.** SNR analyses on the TMS data without- and with task. A) Boxplot showing the signal-to-noise-ratio (SNR) analysis across trials from the TMS session without task using different number of trials (adding 5) each time. B). Boxplot showing the SNR analysis across all trials (N = 36) from the TMS session presented in the main manuscript. No significant statistical differences were founded in between the SNR between number of trials or sessions.

*
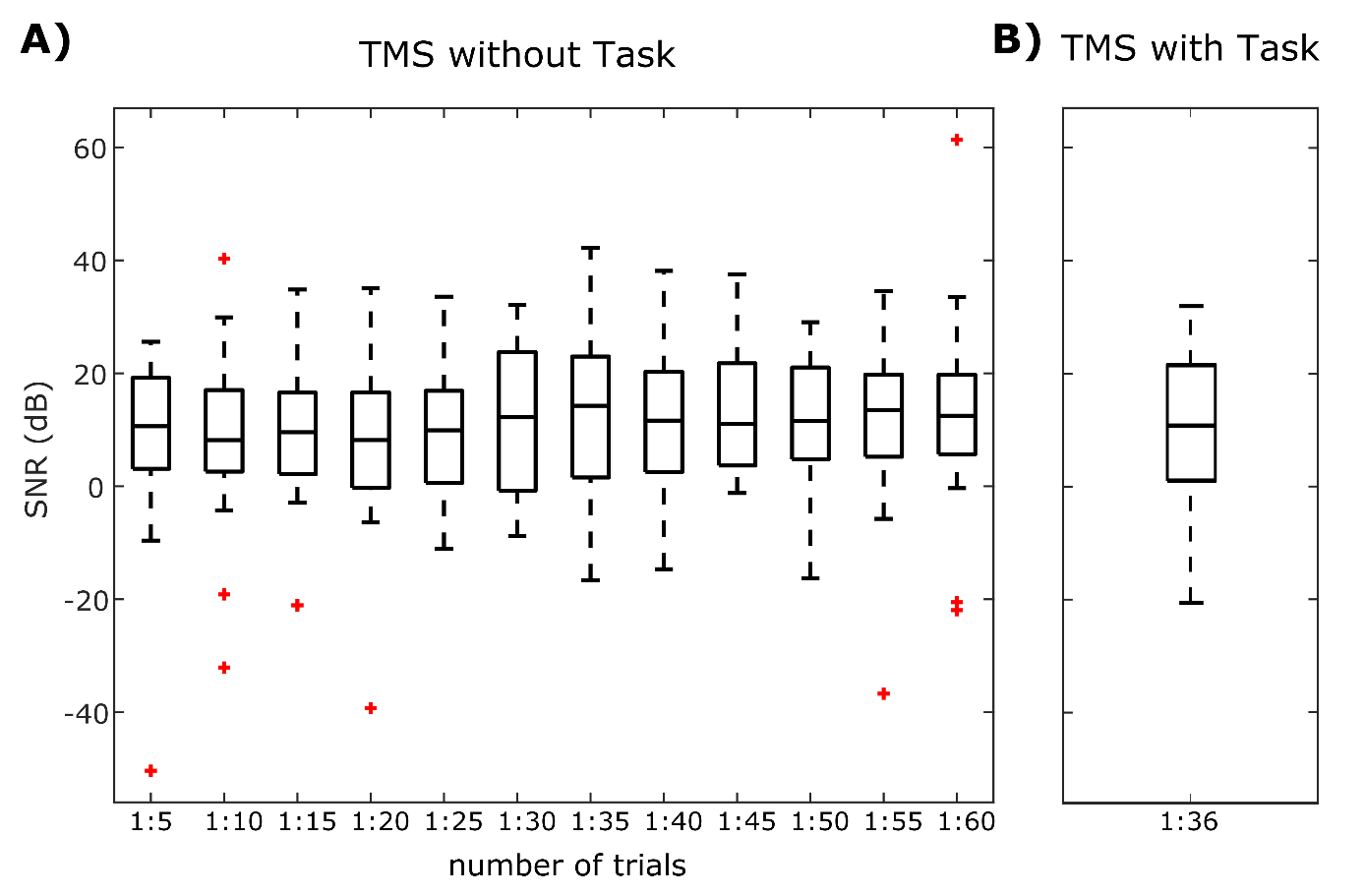
*

**Supplementary Figure 4. TMS-evoked potentials (TEP) for the three different TMS sessions.** Red line = threat TMS-evoked activity; Blue line = no-threat TMS-evoked activity; Grey line = TMS-without-task-evoked activity; the upper solid line shows the significant amplitude differences between TMS-no-task-evoked activity and threat TMS-evoked activity; the upper dotted line shows the significant amplitude differences between TMS-without-task-evoked activity and no-threat TMS-evoked activity.

*
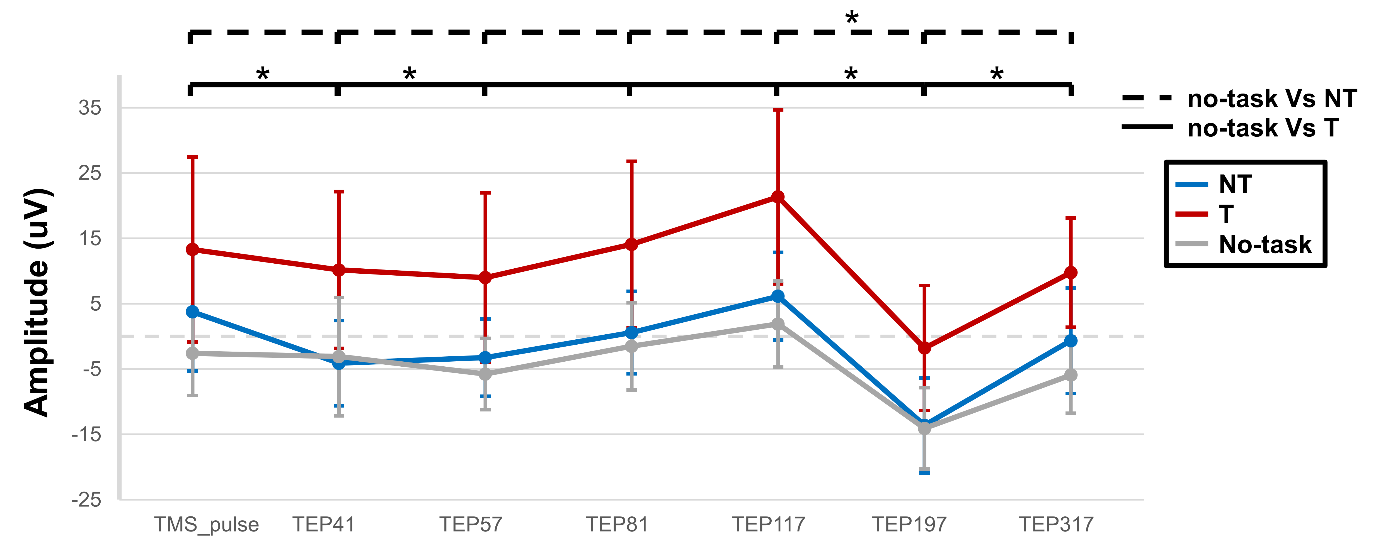
*

**Supplementary Table 1.** Backward step-regression analyses on the sensors grouped by the underlying brain regions. Significant predictions are shown (*p* < 0.05).

| **variable** | **Predicted by** | **r^2^** | **adjusted r^2^** | **F** | ***p*-value** |
| --- | --- | --- | --- | --- | --- |
| **Frontal-LPP** | lh Hp/rh Hp/rh Ins | 0.52 | 0.434 | 5.852 | **0.007** |
| **dmPFC-TEP41** | lh Ins/lh Hp | 0.34 | 0.263 | 4.392 | **0.029** |
| **dmPFC-TEP57** | lh Ins/lh Hp | 0.4 | 0.334 | 5.757 | **0.012** |
| **dmPFC-TEP81** | lh Ins/lh Hp | 0.39 | 0.322 | 5.517 | **0.014** |
| **dmPFC-TEP117** | rh dmPFC/rh Amy/lh Hp/lh Ins | 0.59 | 0.48 | 5.393 | **0.007** |
| **dmPFC-TEP197** | rh dmPFC/rh Amy/lh Hp/lh Ins | 0.77 | 0.711 | 12.66 | **< .001** |
| **dmPFC-TEP317** | rh dmPFC/rh Amy | 0.5 | 0.444 | 8.571 | **0.003** |
| **Frontal-TEP41** | rh dmPFC/rh Amy/lh Ins/lh Hp/rh Hp | 0.64 | 0.505 | 4.882 | **0.009** |
| **Frontal-TEP57** | rh dmPFC/rh Amy/lh Ins/lh Hp/rh Hp | 0.67 | 0.549 | 5.62 | **0.005** |
| **Frontal-TEP81** | rh dmPFC/rh Amy/lh Ins/lh Hp/rh Hp | 0.64 | 0.515 | 5.033 | **0.008** |
| **Frontal-TEP117** | rh dmPFC/rh Amy/lh Ins/lh Hp/rh Hp | 0.66 | 0.536 | 5.387 | **0.006** |
| **Frontal-TEP197** | rh dmPFC/lh Ins/lh Hp | 0.43 | 0.323 | 4.027 | **0.026** |
| **Occipital-TEP41** | rh dmPFC/lh Ins/lh Hp | 0.49 | 0.399 | 5.199 | **0.011** |
| **Occipital-TEP57** | rh dmPFC/lh Ins/lh Hp | 0.43 | 0.322 | 4.002 | **0.027** |
| **Occipital-TEP81** | rh dmPFC/lh Ins/lh Hp | 0.43 | 0.327 | 4.072 | **0.025** |
| **Occipital-TEP117** | rh dmPFC/lh Ins/lh Hp | 0.47 | 0.375 | 4.803 | **0.014** |
| **Occipital-TEP197** | rh dmPFC/rh Amy | 0.48 | 0.413 | 7.685 | **0.004** |
| **Occipital-TEP317** | rh dmPFC/rh Amy/lh Hp | 0.65 | 0.589 | 10.08 | **< .001** |
| **Central-TEP41** | rh dmPFC/rh Amy/lh Hp/lh Ins | 0.59 | 0.486 | 5.484 | **0.006** |
| **Central-TEP57** | rh dmPFC/rh Amy/lh Ins/rh Ins | 0.58 | 0.468 | 5.182 | **0.008** |
| **Central-TEP81** | rh dmPFC/rh Amy/lh Ins/rh Ins | 0.59 | 0.477 | 5.325 | **0.007** |
| **Central-TEP117** | rh dmPFC/rh Amy/lh Ins/rh Ins | 0.62 | 0.514 | 6.018 | **0.004** |
| **Central-TEP197** | rh dmPFC/rh Amy/lh Hp/lh Ins | 0.77 | 0.708 | 12.51 | **< .001** |
| **Central-TEP317** | rh dmPFC/rh Amy/lh Hp/lh Ins | 0.74 | 0.666 | 10.46 | **< .001** |
| **Parietal-TEP41** | lh Ins/lh Hp | 0.38 | 0.301 | 4.735 | **0.015** |
| **Parietal-TEP57** | rh dmPFC/rh Amy/lh Ins/rh Ins | 0.51 | 0.377 | 3.879 | **0.023** |
| **Parietal-TEP81** | rh dmPFC/rh Amy/lh Ins/rh Ins | 0.51 | 0.375 | 3.855 | **0.024** |
| **Parietal-TEP117** | rh dmPFC/rh Amy/lh Hp/lh Ins | 0.58 | 0.467 | 5.167 | **0.008** |
| **Parietal-TEP197** | rh dmPFC/rh Amy | 0.54 | 0.484 | 9.921 | **0.001** |
| **Parietal-TEP317** | rh dmPFC/rh Amy/rh Hp | 0.71 | 0.656 | 13.08 | **< .001** |
| **Temporal-TEP41** | rh dmPFC/rh Amy/lh Hp/lh Ins | 0.66 | 0.57 | 7.299 | **0.002** |
| **Temporal-TEP57** | lh Ins/lh Hp | 0.45 | 0.385 | 6.936 | **0.006** |
| **Temporal-TEP81** | rh dmPFC/rh Amy/lh Hp/lh Ins | 0.61 | 0.509 | 5.916 | **0.005** |
| **Temporal-TEP117** | rh Amy | 0.67 | 0.581 | 7.599 | **0.001** |
| **Temporal-TEP197** | rh dmPFC/rh Amy/lh Hp/lh Ins | 0.74 | 0.665 | 10.44 | **< .001** |
| **Temporal-TEP317** | rh dmPFC/rh Amy/lh Hp/lh Ins | 0.7 | 0.626 | 8.939 | **< .001** |
|  | | | | | |

lh = left hemisphere; rh = right hemisphere; Hp = hippocampus; Ins = insula; Amg = Amygdala; dmPFC = dorsomedial prefonral cortex. Bold numbers indicate significant results (p < 0.05).

**References**

1 Casula, E. P. *et al.* TMS-evoked long-lasting artefacts: A new adaptive algorithm for EEG signal correction. *Clinical neurophysiology : official journal of the International Federation of Clinical Neurophysiology* **128**, 1563-1574, doi:10.1016/j.clinph.2017.06.003 (2017).

2 ter Braack, E. M., de Jonge, B. & van Putten, M. J. Reduction of TMS induced artifacts in EEG using principal component analysis. *IEEE Trans. Neural Syst. Rehab. Eng.* **21**, 376-382 (2013).

3 Kerwin, L. J., Keller, C. J., Wu, W., Narayan, M. & Etkin, A. Test-retest reliability of transcranial magnetic stimulation EEG evoked potentials. *Brain stimulation* **11**, 536-544, doi:10.1016/j.brs.2017.12.010 (2018).

4 Etkin, A., Egner, T. & Kalisch, R. Emotional processing in anterior cingulate and medial prefrontal cortex. *Trends in cognitive sciences* **15**, 85-93, doi:10.1016/j.tics.2010.11.004 (2011).
